# Development and Validation of Predicting Nomograms for Craniopharyngioma: A Retrospective, Multiple-Center, Cohort Study

**DOI:** 10.3389/fonc.2021.691288

**Published:** 2021-07-12

**Authors:** Dingkang Xu, Qingjie Wei, Zhe Li, Yan Hu, Peizhu Hu, Shengqi Zhao, Dengpan Song, Shixiong Lei, Mingchu Zhang, Qiang Gao, Longxiao Zhang, Fangbo Lin, Yuchao Zuo, Xianzhi Liu, Mengzhao Feng, Chunxiao Ma, Fuyou Guo

**Affiliations:** ^1^ Department of Neurosurgery, The First Affiliated Hospital of Zhengzhou University, Zhengzhou, China; ^2^ Department of Neurosurgery, The Third Affiliated Hospital, Zheou University, Zhengzhou, China; ^3^ Department of Pathology, The First Affiliated Hospital of Zhengzhou University, Zhengzhou, China; ^4^ The Department of Cerebrovascular Diseases, The Second Affiliated Hospital of Zhengzhou University, Zhengzhou, China; ^5^ Department of Neurosurgery, Henan Provincial People’s Hospital, Zhengzhou, China

**Keywords:** craniopharyngioma, nomogram, risk stratification, progression-free survival, long-term surveillance, online calculator, neuropsychological status

## Abstract

Craniopharyngiomas (CPs) are benign tumors arising from the sellar region. However, little is known about their clinical features and long-term recurrence due to low morbidity and the lack of large cohort studies. Thus, we aimed to develop nomograms to accurately predict the extent of resection and tumor recurrence using clinical parameters. A total of 545 patients diagnosed with CP between 2009 and 2019 were examined: 381 in the development cohort and 164 in the validation cohort. Least absolute shrinkage and selection operator (LASSO) and Cox regression analyses were performed to establish two nomograms. Receiver operating characteristic (ROC) curves, calibration curves, decision curve analysis (DCA) and Kaplan-Meier (KM) curves were used to evaluate their predictive performance and discriminative power, respectively, in the two cohorts. In addition, the EORTC QLQ-BN20 questionnaire was used to assess neuropsychological status in the follow-up. In the development cohort, the area under the curve (AUC) and C-index were 0.760 and 0.758, respectively, for predicting the extent of resection and 0.78 and 0.75, respectively, for predicting 3-year progression-free survival (PFS) and 5-year PFS. Additionally, the model had a predictive accuracy of 0.785. Both nomograms showed acceptable discrimination in the two cohorts. Moreover, DCA demonstrated excellent clinical benefits from the two nomograms. Finally, participants were classified into two distinct risk groups according to the risk score, and an online calculator was created for convenient clinical use. During long term follow-up, hypothyroidism (77.61%) and hypocortisolism (76.70%) were the most common endocrine dysfunction after surgery and significant deficits were observed concerning visual disorder, motor dysfunction and seizures in the recurrent groups. In particular, better quality of life was associated with gross total resection (GTR), postoperative radiation, anterior interhemispheric (AI) approach and transsphenoidal approach. To our knowledge, these are the first nomograms based on a very large cohort of patients with CP that show potential benefits for guiding treatment decisions and long-term surveillance. The current study demonstrated the online calculator serve as the practical tool for individual strategies based on the patient’s baseline characteristics to achieve a better prognosis.

## Introduction

Craniopharyngiomas (CPs) are benign suprasellar tumors accounting for 2-4% of intracranial tumors (1, 2). Although histologically classified as WHO I tumors, total resection and postoperative management are huge challenges associated with CPs because they are adjacent to vital brain structures, such as the optic chiasm and hypothalamus. Radical resection is considered the first-line treatment because it yields the best overall survival (OS) and progression-free survival (PFS). However, complete resection could lead to increased mortality or poor functional results because of severe endocrine disorders (3). The rate of long-term recurrence could reach an astonishing 58% with or without radiotherapy in the subtotal resection group of some studies (4), so subsequent treatment often becomes an unavoidable problem for such patients. Secondary resection, salvage radiotherapy or intratumoral chemotherapy (5, 6) can be carried out in relapsed patients, though its therapeutic effect is not satisfactory.

In the long-term follow-up, clinical doctors generally pay more attention to the endocrine status of patients than to the neuropsychological status. Only a few studies have focused on these issues. Giese et al. described a cohort of 71 patients with impaired learning performance and short-term memory loss in those with tumor volumes larger than 9 cm^3^ or in those with tumors located in the third ventricle (7). In another center, impaired quality of life and decreased OS were observed in patients with hypothalamus involvement over a minimum 10-year follow-up (8). Nevertheless, there is still a lack of detailed preoperative and postoperative neurological and neurological evaluations in a large cohort.

In recent years, an increasing number of studies have focused on baseline clinical characteristics, such as image features and laboratory tests to predict prognosis and long-term disease recurrence (9, 10). Nomograms, as more friendly and visual tools, have been increasingly used in clinical practice. For instance, the Memorial Sloan Kettering Cancer Center (MSKCC) launched different predictive models to guide clinical treatment more accurately. Although nomograms are extensively used in clinical work, due to the low morbidity associated with CPs, predictive models for CP have not yet been established.

Therefore, this study aimed to develop a novel prognostic model for CP combining preoperative and postoperative features based on two large cohorts. The long-term neuropsychological status of patients was also assessed with the QLQ-BN20 questionnaire. To our knowledge, this is the first study to establish a clinical prediction model and the largest retrospective study of neuropsychological function in CP.

## Materials and Methods

### Patient Selection

**Figure 1:**
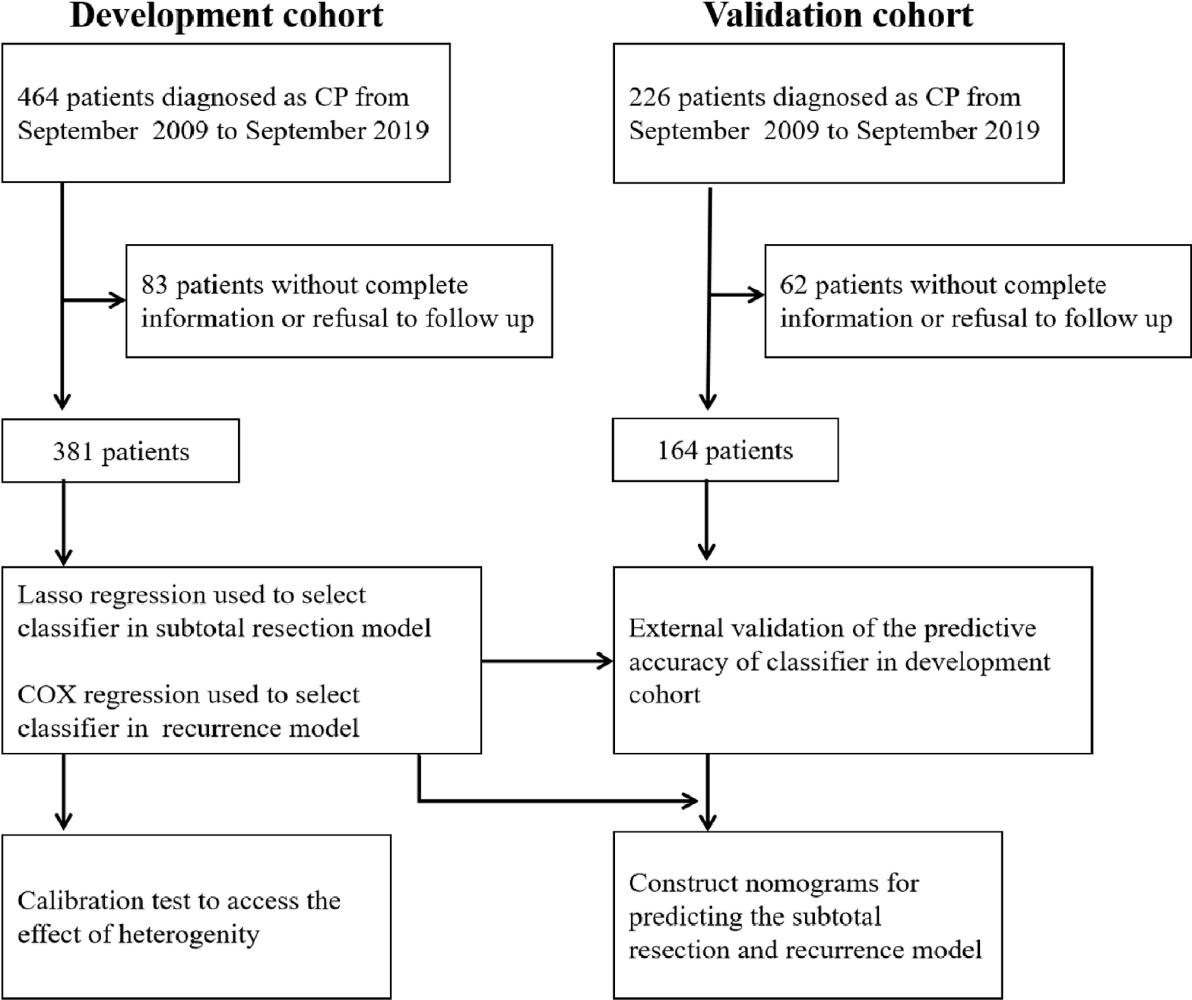
Study cohort.

This retrospective study was approved by the Ethics Committee of Zhengzhou University. All patients gave written informed consent for the surgery and use of data for research purposes prior to the surgery. The protocol was reviewed by the university review board. A total of 545 consecutive patients were diagnosed with CPs and treated at the Department of Neurosurgery, the First Affiliated Hospital of Zhengzhou University, Henan Provincial People’s Hospital and the Third Affiliated Hospital of Zhengzhou University between September 2009 and September 2019. The exclusion criteria were as follows: (1) multiple intracranial tumors with CPs; (2) a history of preoperative adjuvant therapy or surgery in another hospital; (3) death unrelated to disease progression within 1 month after the operation (**Supplemental Table 1**); (4) patients without complete information or refusal to join the study; and (5) malignant CPs. Thus, based on these criteria, 381 patients were included in the development cohort, and 164 patients (7:3) were selected in the validation cohort (**Figure 1**).

### Clinical Parameter Acquisition and Follow-Up Data

All the clinical parameters of patients were recorded and obtained from the hospital electronic medical system. Patients are routinely reviewed at the first 3 months, 1 year, 2 years and 3 years after surgery. Follow-up data were acquired by telephone and in outpatient reviews. Tumor recurrence was assessed by MRI. Endocrinology indicators, including prolactin, luteinizing hormone, follicle stimulating hormone, 24 h ACTH and cortisol, growth hormone, T3, T4 and TSH, were routinely tested in the pre- and postoperative periods.

The operations were performed *via* the pterional approach (n=232), subfrontal approach (n=174), anterior interhemispheric (AI) approach (n=72), transsphenoidal approach (n=47) and transcallosal-interfornix (TI) approach (n=20). The grade of tumor resection was classified as gross total resection (GTR) and subtotal resection (STR). GTR was only assumed when there were no tumor or cystic remnants. CT or MRI reexamination was performed within 1 day of the operation to determine the residual status. Radiotherapy was defined as sequential adjuvant radiotherapy after surgery, excluding salvage therapy when tumor recurrence occurred.

### Image Assessments

All patients routinely underwent MRI or CT upon admission. Moreover, tumor location, hypothalamus and optic nerve involvement, hydrocephalus, calcification and tumor character (Cystic, solid or mix) were obtained from the Picture Archiving and Communication Systems (PACS). The maximum diameter of the tumor was defined as tumor size.

### Quality of Life Questionnaire

The EORTC QLQ-BN20 questionnaire was used to assess health status as previously described (11, 12). Briefly, the QLQ-BN20 consists of four multi-item scales and seven single items, with a high score reflecting severe neuropsychological deficits. The questionnaire was given by telephone and in the outpatient review. Finally, follow-up data were obtained from a total of 317 patients, including 217 patients in the training cohort and 100 patients in the validation cohort.

### Statistical Analysis

All analyses were performed using SPSS version 25 (IBM, Armonk, NY, USA), GraphPad Prism 8.0 and R version 4.0.0 (Package: limma, pheatmap, estimate, ggpubr, e1071, preprocessCore, survival, glmnet, survminer, survivalROC, rms, timeROC). Continuous variables are expressed as the mean ± standard deviation and interquartile range (IQR), while categorical variables are reported as the frequency and percentage. The Shapiro-Wilk test was used to assess the data distribution. Data in normal distribution between two groups were evaluated with the two-tailed Student’s t-test. The Mann-Whitney U and Kruskal Wallis test was used for those that did not conform to a normal distribution. The chi-squared test, Student’s t-test and Kruskal Wallis test with *post hoc* Bonferroni correction were used for data comparison. Then, Kaplan-Meier (KM) survival analyses were used to assess PFS. A log-rank test was used to test the equality of the KM curves.

Depending on the outcome, we first used LASSO or Cox logistics regression analysis to select significant predictors. Then, 5 factors were used to construct two nomograms. Moreover, receiver operating characteristic (ROC) curves, Harrell’s concordance index (C-index), calibration curves and decision curve analysis (DCA) were used to evaluate the predictive performance of the nomograms in both the development and validation cohorts. We further categorized patients into high- and low-risk groups based on their median risk score. Survival analysis was performed to test differences between the two groups. Finally, a risk factor-stratified calculator for long-term recurrence was generated and is available in the additional information online (**Supplementary File**).

## Results

### Patient Characteristics

A total of 545 patients were included in this group of studies: 381 in the training cohort and 164 in the validation cohort. The baseline demographics and clinical characteristics of the patients are listed in **Table 1**. The most frequent symptoms were headaches and visual deficits, which occurred in 256 patients (47.0%) and 258 patients (47.3%), respectively. The mean duration of symptoms to the time of diagnosis was 11.10 ± 18.71 months. The tumor size ranged from 5 mm to 123 mm, with a median diameter of 32 mm, and we changed this factor from a continuous variable to a categorized variable and divided tumor size into 10-mm intervals. A total of 399 (73.2%) patients had varying degrees of hydrocephalus at the preoperative examination. The most common endocrinological deficit before surgery was hypogonadism (273, 50.09%) followed by hypocortisolism (231, 42.39%) and hypothyroidism (230, 42.20%), whereas the percentage of hypogonadism was decreased after surgery (210,38.53%). What’s more, endocrinological dysfunction of corticotropic (418, 76.70%) and thyrotrophic axes (423, 77.61%) were significantly increased in postoperative outcomes (**Table 2**).

**Table 1:** Baseline characteristics of patients in the development and validation cohort.

	Development training cohort (n = 381)	Validation training cohort (n = 164)	Total
**Sex**			
Male	208 (54.6)	89 (54.3)	297 (54.5)
Female	173 (45.4)	75 (45.7)	248 (45.5)
**Age, years**			
Median	37	39	38
IQR	15-52	12.75-61.25	16-52
Range	1-71	2-77	1-77
**Tumor Size, mm**			
Median	32	32	32
IQR	25-42	25-40	25-41
Range	5-123	15-115	5-123
**Duration of symptoms, months**			
0-6	238 (62.5)	105 (64.0)	343 (62.9)
6-12	47 (12.3)	17 (10.4)	63 (11.7)
12-24	46 (12.1)	22 (13.4)	68 (12.5)
>24	50 (13.1)	20 (12.2)	70 (12.8)
**Preoperative endocrine**			
Normal	74 (19.4)	36 (22.0)	110 (20.2)
Abnormal	307 (80.6)	128 (78.0)	435 (79.8)
**Hypothalamus involvement**			
Yes	256 (67.2)	107 (65.2)	363 (66.6)
No	125 (32.8)	57 (34.8)	182 (33.4)
**Optic nerve involvement**			
Yes	340 (89.2)	152 (92.7)	492 (90.3)
No	41 (10.8)	12 (7.3)	53 (9.7)
**Location**			
Intrasellar	14 (3.7)	8 (4.9)	22 (4.0)
Suprasellar	174 (45.7)	60 (36.6)	234 (42.9)
Intrasellar-suprasellar	193 (50.7)	96 (58.5)	289 (53.1)
**Calcification**			
Yes	207 (54.3)	97 (59.1)	304 (55.8)
No	174 (45.7)	67 (40.9)	241 (44.2)
**Hydrocephalus**			
Yes	272 (71.4)	127 (77.4)	399 (73.2)
No	109 (28.6)	37 (22.6)	146 (26.8)
**Features**			
Solid-cystic	295 (77.4)	133 (81.1)	428 (78.5)
Cystic	59 (15.5)	16 (9.8)	75 (13.8)
Solid	27 (7.1)	15 (9.1)	42 (7.7)
**Pathology subtype**			
ACP	312 (81.9)	140 (85.4)	452 (82.9)
PCP	69 (18.1)	24 (14.6)	93 (17.1)
**Surgical approach**			
Pterional approach	163 (42.8)	69 (42.1)	232 (42.6)
Subfrontal approach	124 (32.5)	50 (30.5)	174 (31.9)
AI approach	52 (13.6)	20 (12.2)	72 (13.2)
Transsphenoidal approach	29 (7.6)	18 (11.0)	47 (8.6)
TI approach	13 (3.5)	7 (4.2)	20 (3.7)
**Surgical resection**			
Total resection	204 (53.5)	97 (59.1)	301 (55.2)
Subtotal resection	177 (46.5)	67 (40.9)	244 (44.8)
**Radiotherapy**			
Yes	20 (5.2)	14 (8.5)	34 (6.2)
No	361 (94.8)	150 (91.5)	511 (93.8)
**Recurrence**			
Yes	101 (26.5)	23 (14.0)	124 (22.8)
No	280 (73.5)	141 (86.0)	421 (77.2)
**PFS, months**			
Median	33	32	33
IQR	16-59	12.25-61.75	25.50-60.50
Range	1-115	3-107	1-115

IQR, interquartile range; AI, anterior interhemispheric; TI, transcallosum-interfornix; ACP, Adamantinomatous craniopharyngioma; PCP, Papillary craniopharyngioma.

**Table 2:** Summary of endocrinological in pre and postoperative period.

Endocrinological results before and after surgery					
axis	pre	%	post	%	p
Hypothyroidism	230	42.20%	423	77.61%	p<0.01
Hypocortisolism	231	42.39%	418	76.70%	p<0.01
Hypogonadism	273	50.09%	210	38.53%	p<0.01
GH deficiency	39	7.16%	36	6.61%	p=0.72
Normal	110	20.18%	34	6.24%	p<0.01

**Figure 2:**
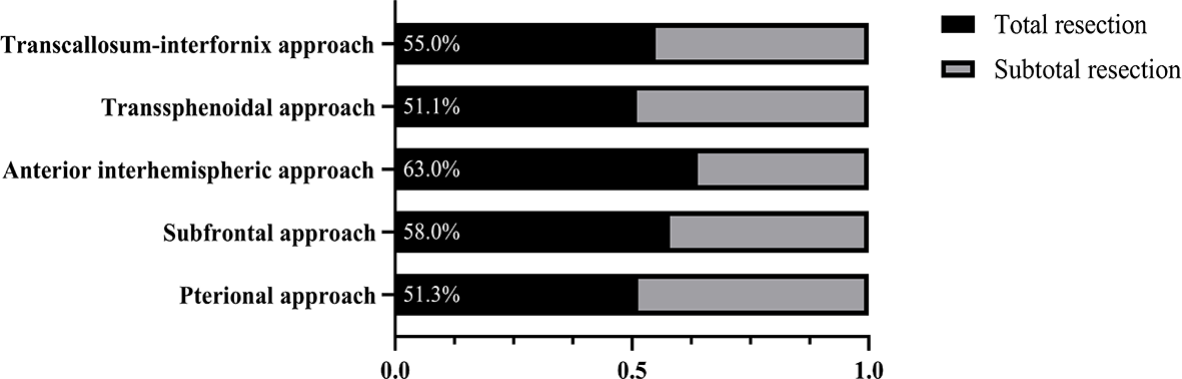
The gross total resection rates of different surgical approaches in CPs.

**Figure 3:**
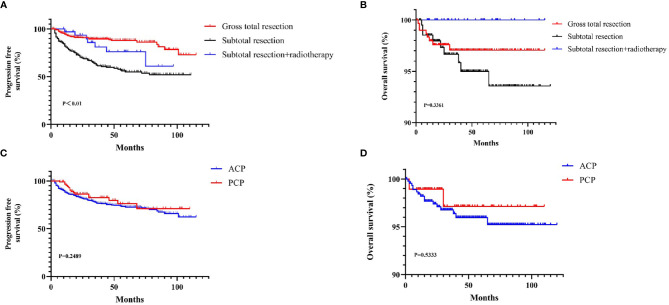
The KM curves for Survival curves for the patients with or without adjuvant treatment **(A, B)** and pathological type **(C, D)**.

The total resection ratio reached 53.5% in the development cohort and 59.1% in the validation cohort. The GTR rates of the different approaches are shown in **Figure 2**; however, there were no significant differences. The mean follow-up time was 40.35 months, and a total of 125 patients experienced tumor recurrence during this period. The cumulative recurrence rates at 1 year, 2 years, 3 years, 5 years and 10 years were 10.8%, 16.0%, 19.3%, 21.3%, and 22.8%, respectively. Finally, 452 adamantinomatous craniopharyngiomas (ACPs, 82.9%) and 93 papillary craniopharyngiomas (PCPs, 17.1%) were enrolled in our study. 292/452 (64.6%) ACPs were accompanied by calcification features, whereas only 12/93 (13.0%) PCPs exhibited calcification features. There was no significant difference in long-term recurrence between these two pathological types (**Figure 3**). In this group, radiotherapy included stereotactic radiotherapy, and 34 patients underwent this treatment after subtotal resection. No malignant transformations were observed in the radiotherapy cohort (34 cases) with a mean follow-up of 43.5 months. There were no differences in long-term recurrence between the GTR and subtotal resection + radiotherapy groups, but radiotherapy significantly improved the PFS rate of patients who underwent subtotal resection (**Figure 3**). No significant differences in overall survival were observed in the extent of resection and pathological type. In addition, initial hydrocephalus had no impact on the extent of resection and PFS.

### Quality of Life

**Figure 4:**
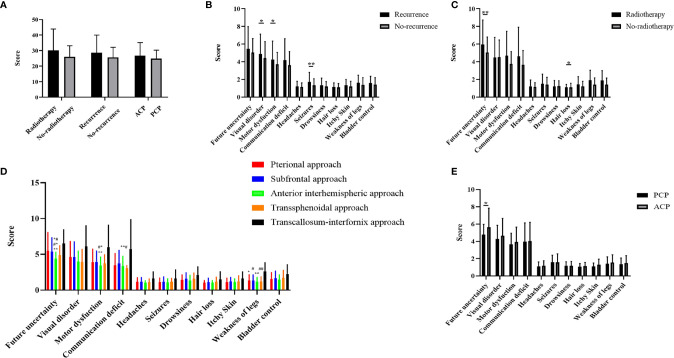
**(A)** Comparison of three groups of variables on the total BN20 score (Mann-Whitney U test, *p < 0.05, **p < 0.01). **(B)** Comparison of craniopharyngioma patient life quality between recurrence and non-recurrence groups (Mann-Whitney U test, *p < 0.05, **p < 0.01). **(C)** Comparison of craniopharyngioma patient life quality between radiotherapy and non-radiotherapy groups (Mann-Whitney U test, *p < 0.05, **p < 0.01). **(D)** Comparison of craniopharyngioma patient life quality between five different approaches (Kruskal Wallis test with *post hoc* Bonferroni correction, p < 0.01, *represent pterional approach *vs*. TI approach, **represent AI approach *vs*. TI approach, ^#^represent subfrontal approach *vs*. TI approach, ^##^represent transsphenoidal approach vs. TI approach, ^*#^represent pterional approach *vs* AI approach, ^#*^represent subfrontal approach vs AI approach, ^**#^represent subfrontal approach *vs* transsphenoidal approach). **(E)** Comparison of craniopharyngioma patient life quality between PCP and ACP groups (Mann-Whitney U test, *p < 0.05).

A total of 317 patients were followed up: 217 in the development cohort and 100 in the validation cohort. The mean total score was 26.30 ± 7.11 in the training cohort and 26.38 ± 9.59 in the validation cohort, and a detailed description is shown in **Table 3**. The total BN20 score was not significantly different according to radiotherapy, relapse groups or pathological type, however difference were observed concerning different items (p<0.05). Significant differences in visual disorder (5.00 ± 2.34 *vs* 4.36 ± 1.94, p<0.05), motor dysfunction (4.34 ± 2.25 *vs* 3.74 ± 1.42, p<0.05) and seizures (1.38 ± 0.86 *vs* 1.10 ± 0.48, p<0.01) were observed between the recurrence cohort and the other cohorts. Patients who received radiotherapy had higher scores related to future uncertainty (6.46 ± 3.04 *vs* 5.13 ± 1.94, p<0.01) and hair loss (1.27 ± 0.60 *vs* 1.10 ± 0.44, p<0.05) than patients who did not receive radiotherapy. In addition, scores related to future uncertainty in ACP was higher than that in PCP(5.39 ± 2.23 *vs* 4.66 ± 1.20, p<0.05). We further explored the impact of different approaches on terminal BN20 scores. The patients in subfrontal approach and pterional approach had higher scores related to future uncertainty versus AI approach (5.49 ± 2.56 *vs* 4.38 ± 0.90, 5.36 ± 1.95 *vs* 4.38 ± 0.90, p<0.01); The BN20 scores related to weak of legs with the pterional approach, subfrontal approach, AI approach, and transsphenoidal approach versus the TI approach were 1.35 ± 0.80, 1.36 ± 0.76, 1.21 ± 0.59, and 1.23 ± 0.63 *vs* 2.63 ± 1.19, respectively (p<0.01, **Figure 4**). Moreover, the TI approach demonstrated higher scores than the AI approach concerning future uncertainty and motor dysfunction (4.38 ± 0.90 *vs* 6.50 ± 1.93 and 3.43 ± 1.19 *vs* 6.00 ± 3.07, p<0.01, **Figure 4**). Detailed description and P value were shown in supplemental material.

**Table 3:** BN20 scores of patients in the development and validation cohort.

	Development training cohort (n = 217)	Validation training cohort (n = 100)	Total
Future uncertainty	5.08 ± 1.61	5.29 ± 2.28	5.15 ± 1.85
Visual disorder	4.55 ± 1.92	4.47 ± 2.08	4.53 ± 1.97
Motor dysfunction	3.84 ± 1.44	3.83 ± 1.83	3.84 ± 1.57
Communication deficit	3.67 ± 1.64	3.89 ± 2.16	3.74 ± 1.82
Headaches	1.18 ± 0.54	1.10 ± 0.41	1.15 ± 0.50
Seizures	1.45 ± 0.88	1.38 ± 0.79	1.43 ± 0.85
Drowsiness	1.25 ± 0.63	1.22 ± 0.58	1.24 ± 0.61
Hair loss	1.11 ± 0.43	1.12 ± 0.48	1.11 ± 0.44
Itchy skin	1.25 ± 0.62	1.21 ± 0.59	1.24 ± 0.61
Weakness of legs	1.47 ± 0.86	1.39 ± 0.75	1.44 ± 0.83
Bladder control	1.45 ± 0.74	1.48 ± 0.89	1.46 ± 0.79
Total scores	26.30 ± 7.11	26.38 ± 9.59	26.33 ± 7.96

### Nomogram Construction and External Validation

**Figure 5:**
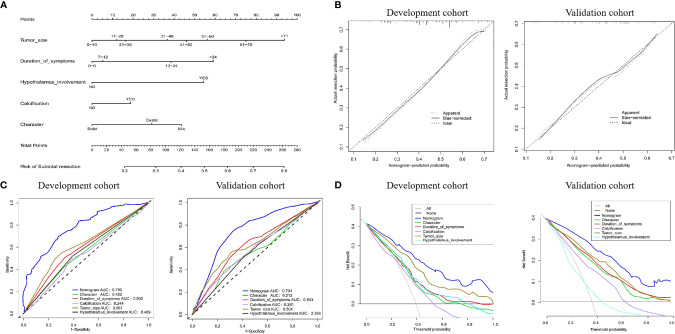
**(A)** Nomogram for predicting the extent of resection. For each variable, a verticle line was drawn to the upward point line and the total score of 6 variables could be obtained to calculate the risk of subtotal resection. **(B)** Calibration curves for development and validation cohort. **(C)** The ROC curves of the nomogram, tumor size, duration of symptoms, hypothalamus involvement, calcification, character and combined nomogram in the development and validation cohort. **(D)** The decision curve analysis of the nomogram. The Y-axis represents the net benefit. The larger net benefit means a patient can obtain the more benefit of the model.

**Figure 6:**
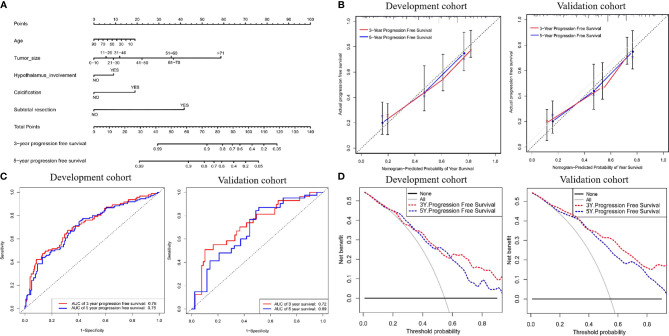
**(A)** Nomogram for predicting the probability of 3- and 5-year progression free survival. For each variable, a verticle line was drawn to the upward point line and the total score of 6 variables could be obtained to calculate the probability of progression free survival. **(B)** Calibration curves for development and validation cohort. **(C)** The ROC curves of the nomogram, age, tumor size, hypothalamus involvement, calcification, character and combined nomogram in the development and validation cohort. **(D)** The decision curve analysis of the nomogram. The Y-axis represents the net benefit. The larger net benefit means a patient can obtain the more benefit of the model.

Based on the LASSO and Cox regression models, two nomograms that integrated significant predictors were generated, as demonstrated in **Figures 5** and **6**. The nomograms used to predict the extent of resection compromised tumor size, duration of symptoms, hypothalamus involvement, calcification and characteristics. As shown in **Figure 5**, the AUCs of individual tumor size, duration of symptoms, hypothalamus involvement, calcification and characteristics were 0.587, 0.502, 0.489, 0.244 and 0.422, respectively, but the combination nomogram reached an AUC of 0.760 (95% CI: 0.73-0.78), with a C-index of 0.758 (95% CI: 0.721-0.793). To further verify the efficacy of the nomogram, we investigated the model with the validation cohort. The AUC of the external cohort was 0.704 (95% CI: 0.68-0.74), with an AUC that was obviously lower than that of other individual predictors. The calibration curve yielded agreeable results. DCA showed more favorable clinical applications with the nomograms than with individual predictors, demonstrating the feasibility of the nomograms for making valuable judgments on prognosis.

To better predict long-term recurrence during follow-up, nomograms involving age, tumor size, hypothalamus involvement, calcification and subtotal resection were generated. The AUCs of the 3- and 5-year PFS nomograms were 0.78 (95% CI: 0.72-0.83) and 0.75 (95% CI: 0.69-0.80, C-index: 0.785, 95% CI: 0.757-0.809), respectively; the AUCs in the validation cohort reached 0.72 (95% CI: 0.69-0.76) and 0.69 (95% CI: 0.65-0.72, C-index: 0.735, 95% CI: 0.703-0.75), respectively. The calibration curve was favorable. Moreover, DCA was performed in both cohorts (**Figure 6**).

### Performance of the Nomogram in Stratifying Patient Risk

**Figure 7:**
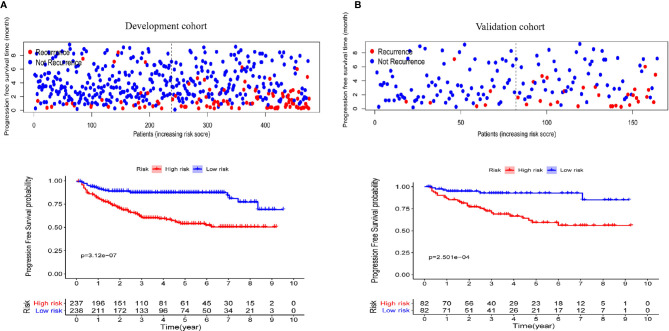
The KM curves for high-risk group (red) and low-risk group (blue) in the development **(A)** and validation cohort **(B)**.

**Figure 8:**
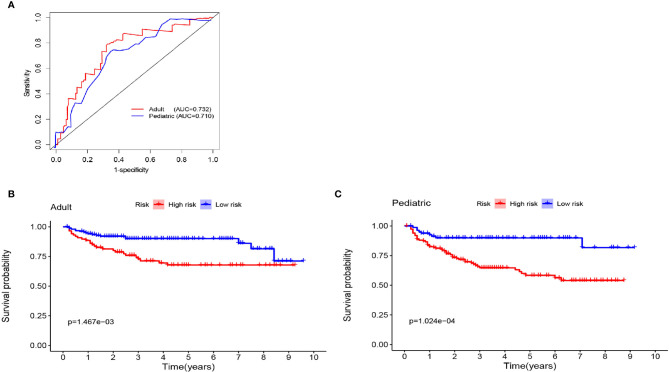
**(A)** Nomogram for predicting the extent of resection in adult and pediatric cohort. The KM curves for high-risk group (red) and low-risk group (blue) in the adult **(B)** and pediatric cohort **(C)**.

To better distinguish the patients, we further stratified individuals into high- and low-risk groups based on their median risk score. The hazard ratios (HRs) and 95% confidence intervals (CIs) of the high-risk groups in the training and validation cohorts were 1.67 (95% CI: 1.04-2.58, p<0.01) and 1.41 (95% CI:1.18-2.14, p<0.01), respectively (compared to the low-risk groups, **Figure 7**). Moreover, as shown in **Figure 7**, PFS in the first three years decreased significantly faster than that over the following years. In addition, the AUCs of the adult and pediatric nomograms were 0.732 (95% CI: 0.711-0.753) and 0.710 (95% CI: 0.693-0.727), respectively. We further verified the reliability of predicting long-term recurrence in the adult and pediatric groups using KM curve analysis. The hazard ratios (HRs) 95% and confidence intervals (CIs) of the high-risk groups in the adult and pediatric cohorts were 1.59 (**Figure 8**, 95% CI: 1.32-1.86, p<0.01) and 1.41 (95% CI:1.42-1.84, p<0.01). An online calculator is available at https://dingkangxu.github.io/Predicting_tool_for_craniopharyngioma/.

## Discussion

Although mounting studies and abundant clinical experience could guide neurosurgeons to perform skilled resection of CPs, an accurate assessment of the characteristics of each patient during the perioperative period remains necessary for individualized treatment. In this study, we developed a new clinical decision-making system to predict the resection and long-term recurrence risks of CP based on a large cohort and validated it on a cohort from another hospital. This system might allow clinicians to make more informed therapeutic decisions regarding adjuvant treatment. In addition, there were some striking features obtained from the present series of patients: 1. the risk of long-term recurrence in the high-risk group was significantly higher than that in the low-risk group; 2. the risk of recurrence among all patients was 19% within the first three years of surgery and then entered a plateau; 3. there was no difference in the BN20 score according to sex or pathological subtype; however, the radiotherapy and recurrence groups demonstrated higher scores than the other groups; 4. there was no significant difference in PFS between the GTR and STR+ radiotherapy groups, but radiotherapy prolonged PFS in the STR group; 5. the transcallosal fornix approach was associated with poor neuropsychological function, whereas the transsphenoidal approach exhibited better performance; and 6. hypothalamus involvement was a strong indicator in predicting both the grade of resection and long-term recurrence. To our knowledge, this is the first time predicting tools have been reported in CPs. The risk classification of patients and imaging reexamination plan based on these nomograms for postoperative follow-up and long-term surveillance will greatly benefit the clinical work.

In general, GTR should be pursued for the treatment of CP because it provides a chance for clinical cure, whereas radical treatment might cause catastrophic hypothalamic damage, especially in children. Different surgical approaches applied in the management of CP depend on tumor location, the growth pattern and the surgeon’s experience (13) since there was no difference in the grade of resection between surgical approaches in our cohort. Based on our experience, strict indications should be required for the transnasal approach (14) (e.g., tumors purely in the sellar region). The ideal approach should maximize total tumor resection and maintain high-quality survival after surgery. Currently, an increasing number of approaches are being used in the resection of CPs (15), but a large study is needed to verify the results. It was reported Liu et al. proposed a novel QST classification for understanding the growth pattern of tumors and could be used to guide surgical procedures (16). Considering the current medical environment in China and the risks brought by radical resection, in particular, the expectations of patients may be influenced by the local culture. We believe that it is necessary to objectively predict the degree of resection based on clinical features before surgery. Of note, predictive tools cannot replace intraoperative judgments about the possible degree of resection. Thus, the predictive model can become an important and timely communication tool between doctors and patients and allow patients to better understand the risks of surgery from the doctor’s perspective.

In the past, several independent risk factors related to tumor recurrence, such as age, extent of resection, hypothalamus involvement, and postoperative radiotherapy, have been reported (1, 2), but what does the proportion of these factors play in tumor recurrence? Therefore, identification of the risk of recurrence in patients with CP is crucial for personalized treatment planning. Here, we established a model that can accurately predict long-term tumor recurrence. De Vile C J et al. described an age less than 5 years as a significant predictive factor for recurrence, which might be due to the difficulty associated with giving adjacent radiotherapy to children and its side effects on cognitive function (17). To date, the prognostic value of pathological subtype remains controversial, with some viewpoints suggesting that PCP has a better prognosis than ACP (18). In our study. ACP and PCP were not different according to the KM survival curves (**Figure 3**). At present, some surgeons believe that total resection can achieve the best therapeutic effect and that subtotal resection + radiotherapy does not improve prognosis and might even result in the side effects observed with radiotherapy (19–23); however, radiotherapy can significantly prolong the PFS period following incomplete resection, similar positive effect of radiotherapy were observed in previous literatures (19). Patients were stratified by risk scores into high- and low-risk groups based on the median risk scores to scientifically guide long-term follow-up in the clinic and more efficiently monitor tumor recurrence and reduce time and economic cost. The first three years after the operation was the peak period of tumor recurrence, after which it entered a plateau period, and only a few patients relapsed at this time. Based on the prediction tool and the plateau, we strongly recommend that patients at high risk undergo MRI at least every 6 months for 3 years after surgery. There are some points that need to be considered when applying the model. For the study of baseline data, we tried to avoid bias that may have been caused by subjective factors, such as operative time and amount of bleeding. Although this may reduce the coverage of the model, it would be more suitable for clinical practice. Finally, calcification in the CT scan was not always in accordance with intraoperative manipulation, suggesting that a thin-layer CT scan should be performed as much as possible to determine the authentic situation and to improve the efficiency of the two nomograms.

With the development of neurosurgery technology, total resection of CP can obtain long-term OS. The latest viewpoints have gradually transformed CP from a curative tumor to a chronic disease (24), so its neuropsychological outcome it has been noted in recent years. A large proportion of patients cannot return to work because of the endocrine disorders and mental deficits remaining after surgery (7). Considering the *post hoc* LSD test may show false positive results whereas the Bonferroni correction is conservative (0.5 *vs* 0.05) in the evaluation of neuropsychological function, p<0.01 was used as the threshold for the pairwise comparison. We have attached the original P value to **Supplementary Material**. In our cohort, radiotherapy was associated with higher risks concerning hair loss, consistent with our understanding (25). Regarding the surgical approach, the BN20 scores were better with the AI approach than with the TI approach. On the one hand, the latter could be explained by its closer location to the third ventricle, larger size, and requirement for an incision in the corpus callosum. On the other hand, the AI approach could reduce the stretching of brain tissue. Moreover, the transsphenoidal approach showed better quality of life in terms of long-term neurological function after surgery. When using the transsphenoidal approach, the surgeon needs to be aware of the catastrophic consequences of vascular disturbances during the operation. Unfortunately, this study focused only on function at the last postoperative follow-up. A detailed functional assessment both pre- and postoperatively is necessary in the future.

However, there are a few limitations to this research. Although two nomograms were developed and validated in three large centers, our study was retrospective, with inevitable limitations. Furthermore, some data, such as BMI and morbid obesity were lacking during the follow-up. Thus, our results need to be further validated in a prospective cohort and in other populations.

## Conclusion

In conclusion, we developed novel nomograms to better predict the extent of resection before surgery and the risk of long-term recurrence; thus, these nomograms might allow neurosurgeons and patients to benefit from clinical care and decision making. Finally, we developed an online calculator to stratify patients into different risk groups to increase the utility of follow-up surveillance and management strategies. As the first nomogram with external validation based on a large series, we believe that these two predictive tools will provide guidance in individual treatment and clinical applications.

## Data Availability Statement

The raw data supporting the conclusions of this article will be made available by the authors, without undue reservation.

## Ethics Statement

Written informed consent was obtained from the individual(s), and minor(s)’ legal guardian/next of kin, for the publication of any potentially identifiable images or data included in this article.

## Author Contributions

DX and FG: conception and design, analysis, data collection, and manuscript writing. QW, PH, YH, YZ, and MF: data collection, analysis, and manuscript reviewing. QW, ZL, SZ, DS, SL, MZ, QG, LZ, and FL: data collection and analysis. FG, XL, and CM: manuscript reviewing. All authors contributed to the article and approved the submitted version.

## Funding

This work was supported by grants from the National Natural Science Foundation of China (U1204807), the Science and Technology Department of Henan Province (192102310113), the Medical Science and Technique Foundation of Henan Province (SB201901007), and the Educational Department of Henan Province (19B320017).

## Conflict of Interest

The authors declare that the research was conducted in the absence of any commercial or financial relationships that could be construed as a potential conflict of interest.

## Supplementary Material

Supplementary Table 1 Excluded patients in postoperative period.
