# Supplementary material for: Development and Validation of Predicting Nomograms for Craniopharyngioma: A Retrospective, Multiple-Center, Cohort Study
Source: Front Oncol. 2021 Jul 12;11:691288. doi: 10.3389/fonc.2021.691288 (PMC8312552; doi:10.3389/fonc.2021.691288)
Supplement: Supplementary Table 1 — Excluded patients in postoperative period. [file Table_1.doc]

Supplemental table 1：Excluded patients in postoperative period.

| Patients | Gender | Age (years) | Cause of death |
| --- | --- | --- | --- |
| Case 1 | Female | 2 | Hypothalamic damage |
| Case 2 | Female | 53 | Intracerebral hemorrhage |
| Case 3 | Female | 54 | Pulmonary embolism |
| Case 4 | Male | 37 | Hypothalamic damage |
| Case 5 | Female | 38 | Intracerebral hemorrhage |
| Case 6 | Male | 39 | Pulmonary embolism |
