# Supplementary figures and images for: Development and Validation of Predicting Nomograms for Craniopharyngioma: A Retrospective, Multiple-Center, Cohort Study
Source: Front Oncol. 2021 Jul 12;11:691288. doi: 10.3389/fonc.2021.691288 (PMC8312552; doi:10.3389/fonc.2021.691288)

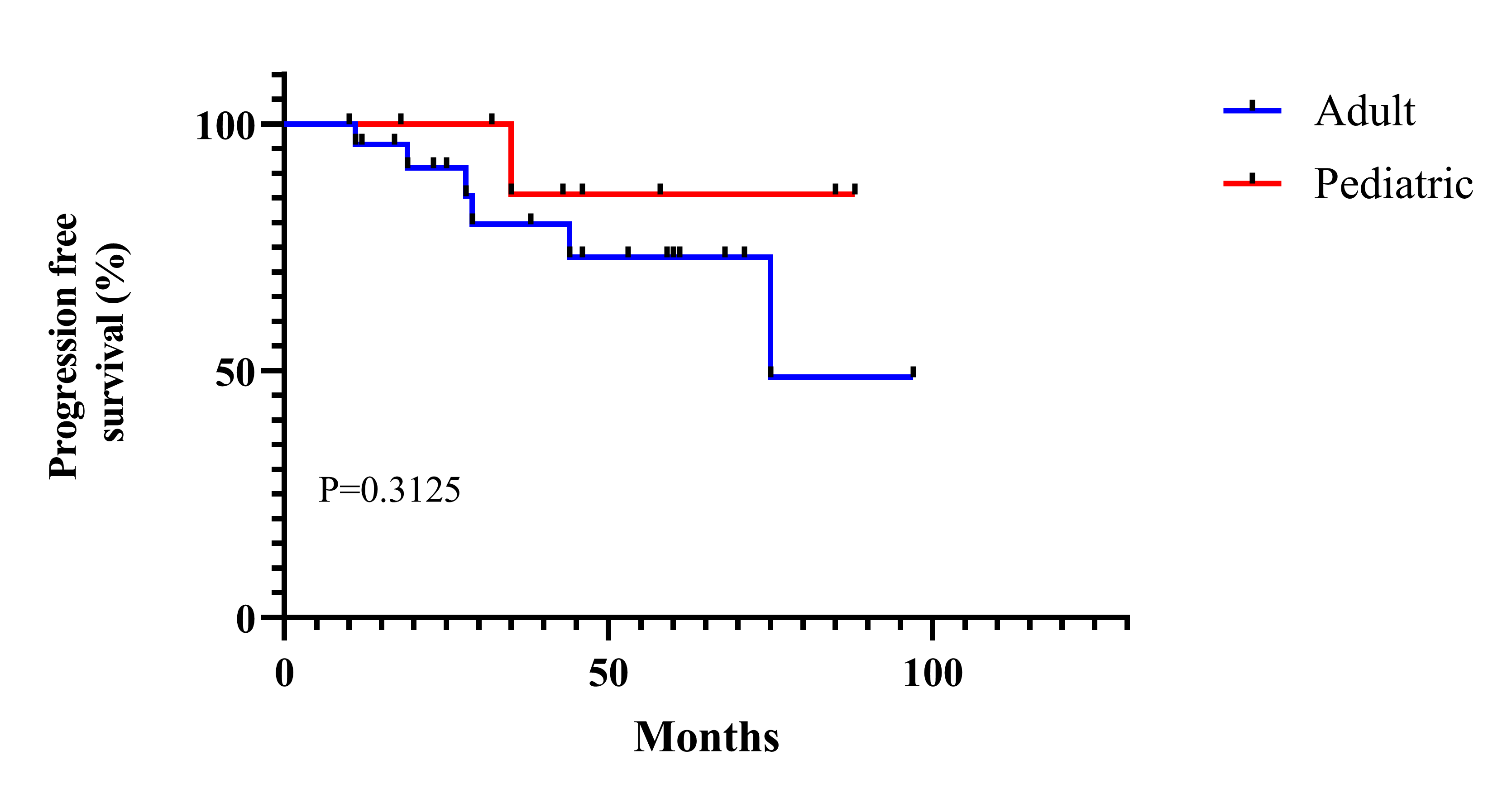

Supplement: Supplementary file 2 [file Image_1.tif]
